# Establishment and drug screening of patient-derived extrahepatic biliary tract carcinoma organoids

**DOI:** 10.1186/s12935-021-02219-w

**Published:** 2021-10-02

**Authors:** Zhiwei Wang, Yinghao Guo, Yun Jin, Xiaoxiao Zhang, Hao Geng, Guangyuan Xie, Dan Ye, Yuanquan Yu, Daren Liu, Donger Zhou, Baizhou Li, Yan Luo, Shuyou Peng, Jiangtao Li

**Affiliations:** 1 Department of Surgery, The Second Affiliated Hospital, Zhejiang University School of Medicine, Hangzhou, 310009 Zhejiang China; 2 Department of Pathology, The Second Affiliated Hospital, Zhejiang University School of Medicine, Hangzhou, 310009 Zhejiang China; 3 Department of Biochemistry and Cancer Institute of the Second Affiliated Hospital (Key Laboratory of Cancer Prevention and Intervention of China National MOE), Zhejiang University School of Medicine, Hangzhou, Zhejiang China; 4 Department of Pharmacy, Tongde Hospital of Zhejiang Province, Hangzhou, Zhejiang China

**Keywords:** Extrahepatic biliary tract carcinoma, Organoids, Pathological staining, Genetic profiles, Drug screening

## Abstract

**Background:**

Patient-derived organoids (PDO) have been proposed as a novel in vitro method of drug screening for different types of cancer. However, to date, extrahepatic biliary tract carcinoma (eBTC) PDOs have not yet been fully established.

**Methods:**

We collected six samples of gallbladder carcinoma (GBC) and one sample of extrahepatic cholangiocarcinoma (eCCA) from seven patients to attempt to establish eBTC PDOs for drug screening. We successfully established five GBC and one eCCA PDOs. Histological staining was used to compare structural features between the original tissues and cancer PDOs. Whole exome sequencing (WES) was performed to analyze the genetic profiles of original tissues and cancer PDOs. Drug screening, including gemcitabine, 5-fluorouracil, cisplatin, paclitaxel, infigratinib, and ivosidenib, was measured and verified by clinical effects in certain cases.

**Results:**

Different PDOs exhibited diverse growth rates during in vitro culture. Hematoxylin and eosin staining demonstrated that the structures of most cancer PDOs retained the original structures of adenocarcinoma. Immunohistological and periodic acid-schiff staining revealed that marker expression in cancer PDOs was similar to that of the original specimens. Genetic profiles from the four original specimens, as well as paired cancer PDOs, were analyzed using whole exome sequencing. Three of the four PDOs exhibited a high degree of similarity when compared to the original specimens, except for GBC2 PDO, which only had a concordance of 74% in the proportion of single nucleotide polymorphisms in the coding sequence. In general, gemcitabine was found to be the most efficient drug for eBTC treatment, as it showed moderate or significant inhibitory impact on cancer growth. Results from drug screening were confirmed to a certain extent by three clinical cases.

**Conclusions:**

Our study successfully established a series of eBTC PDOs, which contributed to the field of eBTC PDOs. Additional enhancements should be explored to improve the growth rate of PDOs and to preserve their immune microenvironment.

**Supplementary Information:**

The online version contains supplementary material available at 10.1186/s12935-021-02219-w.

## Background

Extrahepatic biliary tract carcinoma (eBTC), which comprises extrahepatic cholangiocarcinoma (eCCA) and gallbladder carcinoma (GBC), is a relatively rare malignancy that occurs in the digestive system. The incidence of eCCA is similar to that of intrahepatic cholangiocarcinoma but lower than that of perihilar cholangiocarcinoma [1]. The risk factors for eCCA include primary sclerosing cholangitis, smoking, and heavy alcohol drinking [2, 3]. In contrast, GBC is one of the most common malignancies of the digestive system, accounting for approximately 1% of all cancers and 80–95% of all eBTCs [4]. The main causes of GBC are gallstones and chronic cholecystitis and can therefore easily be confused with them due to similar symptoms [5]. To date, surgical resection remains to be the most efficient treatment for eBTC. However, fewer than 50% of GBC patients and 35–68% of eCCA patients are eligible for surgical resection [5–7]. Early eCCA and GBC are hard to detect due to a lack of specific symptoms. Patients with eCCA are often diagnosed owing to jaundice and recurrent abdominal pain; however, most tumors are already developed to an advanced stage at the moment of diagnosis. Although traditional chemotherapy and targeted therapy have been used in most eBTC patients, these treatments have not yet been proven to be beneficial for the prognosis and survival of patients due to drug resistance and recurrence [8]. Novel in vitro methods that detect the sensitivity of eBTC to drugs have been developed in recent years, including the establishment of patient-derived organoids (PDOs).

Cells in traditional two-dimensional (2D) culture systems form a monolayer morphology and lack normal contact with each other, which makes it nearly impossible to mimic essential characteristics of cells in vivo. Thus, three-dimensional (3D) culture systems were developed to address the defects of the 2D culture systems. PDO is a type of in vitro 3D culture system, which utilizes Matrigel or other protein or collagen matrix as scaffold for cell growth to form a 3D structure, as well as provides certain cytokines necessary for tumor growth to maintain the specific structure and morphology of tumor. It is superior to traditional 2D culture system in its ability to maintain physiological structure and genetic profiles of the original tissue. Cancer PDOs, also known as tumoroids, have been confirmed to highly resemble the original tissues in structure and genetic profiles [9–12]. Additionally, previous studies have shown that cancer PDOs are efficient at detecting drug sensitivity of the original cancer [9–15]. For instance, Broutier et al. reported that cancer PDO allowed for the identification of drug sensitivities across primary hepatocellular carcinoma [14]. Ganesh et al. also reported that cancer PDO of rectal cancer can predict the sensitivity of original cancer to both chemotherapy and radiotherapy [15]. Recently, our team has successfully established PDOs of intrahepatic cholangiocarcinoma (iCCA) and evaluated its drug sensitivity, which was consistent with the observed clinical effects [16]. However, very few studies have established PDO from eBTC to date. Saito et al. reported the establishment and drug screening of PDO for BTC [11]. However, their study only included three PDOs of iCCA and one PDO of GBC, and the results from the drug screening were not corroborated by additional cases.

Considering the potential value of cancer PDOs in the treatment of eBTC, we aimed to establish a series of PDOs from eBTC to determine whether the physiological structure and genetic profiles are maintained in these cultures. Additionally, we performed a drug screening using PDO, the results of which were confirmed in several clinical cases.

## Methods

### Specimen collection

This study was granted approval by the ethics committee of the Second Affiliated Hospital, Zhejiang University, School of Medicine (No. 2019-408) and conducted in compliance with the ethical principles of the Declaration of Helsinki. Written informed consents of participants were obtained prior to surgery. One eCCA specimen and six GBC specimens were collected after surgical resection. After collection, the specimens were promptly transferred to the laboratory on ice (within 4 h).

### Culture of PDOs

Collected specimens were initially washed two or three times using sterile normal saline and sliced into 0.5–1 mm^3^ pieces using ophthalmic scissors. These pieces were then digested using 1.5 mg/mL of collagenase D (Roche, Basel, Switzerland) for at least three hours at 37 °C, and were blown once every hour. Dissociated cells were filtered through a 100-µm cell strainer, when specimens were digested into single cancer cells or small cluster of cancer cells. The number of resulting cancer cells was counted, centrifuged, and pelleted at 300×*g* for 5 min at room temperature.

Growth factor reduced Matrigel matrix (Corning, NY, USA) was utilized as skeleton for the PDO culture. After being stored at − 20 °C and thawed on ice, the matrix was mixed with complete culture medium at a ratio of 1:1 and 200 µL of the mixture were used for coating a 24-well plate in advance. Cell pellets were resuspended in the mixture at a concentration of approximately 5 × 10^5^ cells/mL. Then, 200 µL of cell suspension were seeded onto the pre-coated wells and 500 µL of complete culture medium was added to each well for culture, which was replaced every 4 days. The PDO culture was completed after two weeks or when the diameters of most PDO had reached 150 μm.

The complete culture medium included advanced Dulbecco’s modified Eagle medium/F12 (Gibco, CS, USA) supplemented with penicillin/streptomycin (1×, ThermoFisher, MA, USA), Glutamax (1×; ThermoFisher), B27 supplement (1×; Gibco, CS, USA), N2 supplement (1×; Gibco, CS, USA), HEPES (10 mM, ThermoFisher, MA, USA), gastrin (10 nM; Sigma Aldrich, MO, USA), A83-01 (5 µM; Tocris, Bristol, UK), Y-27,632 (10 µM; Tocris, Bristol, UK), recombinant human epidermal growth factor (50 ng/mL; PeproTech, NJ, USA), recombinant human fibroblast growth factor 10 (100 ng/mL; PeproTech), recombinant human R-Spondin1 (500 ng/mL; PeproTech,), recombinant human Noggin (100 ng/mL; PeproTech), and Afamin/Wnt3a CM (10% v/v; MBL Life Science, TKY, Japan).

### Histology and staining

After completing the culture of PDOs, the Matrigel matrix was digested using 1.5 mg/mL dispase II (Roche, Basel, Switzerland) for 1 h at 37 °C and the PDOs were pelleted by centrifugation of 300×*g* for 5 min at room temperature. These pellets were then divided in three and one part of each pellet was fixed in 10% neutral-buffered formalin for 2–4 h. Afterwards, these PDO pellets were resuspended evenly in 4% low-melting agarose after centrifugation. After low-melting agarose solidified at 4 °C, the fixed original cancer specimens along with low-melting agarose containing PDOs were dehydrated and embedded into paraffin blocks. Next, 4-µm thick sections of the paraffin blocks were prepared for routine hematoxylin and eosin (H&E) and immunohistological staining.

Mouse monoclonal antibodies targeting cytokeratin-7 (CK7; Abcam, Cambridge, UK, 1:2000) mucin-1 (MUC1; Abcam, Cambridge, UK, 1:500) and epithelial cell adhesion molecule (EpCAM Abcam, Cambridge, UK, 1:500) were utilized as primary antibodies, while 3,3′-diaminobenzidine was used as a chromogenic agent for immunohistological staining. Yellow, light brown, and dark brown staining represented by weak, moderate, and strong expression, respectively. In addition, we performed periodic acid-schiff (PAS) staining to assess the distribution of glycogen in original cancer specimens as well as PDOs. Red or pink staining evidenced the degree of distribution of glycogen.

### Whole exome sequencing (WES)

One third of each PDO pellet was used for total DNA extraction from the original cancer specimens and PDOs using GenElute Mammalian Genomic DNA miniprep kits (Sigma Aldrich), according to the manufacturer’s instructions. Degradation and RNA contamination in the extracted DNA were determined using 1% agarose gels. The total concentration of extracted DNA was quantified using the Qubit DNA Assay Kit in a Qubit 3.0 Fluorometer (Invitrogen, CA, USA). The Agilent liquid capture system (Agilent SureSelect Human All Exon V6) was utilized for the enrichment of exome sequences from 0.4 µg genomic DNA, according to the manufacturer’s instructions. The genomic DNA was randomly fragmented into 180–280 bp, end repaired, and phosphorylated. The DNA fragments with ligated adapter molecules were then enriched within a PCR reaction. The magnetic beads were then used for capturing gene exons. The captured libraries were enriched using PCR to add index tags for preparation of sequencing. After generating clusters of index-coded samples, DNA libraries were sequenced on the Illumina platform. SAMtools were utilized for variant calling and single nucleotide polymorphisms (SNPs) and insertions and deletions (InDels) identification. Control-FREEC was used for the detection of copy number variations (CNVs). The somatic single nucleotide variations (SNVs), InDel, and CNV were detected using muTect, Strelka, and Control-FREEC, respectively.

### Drug screening

Briefly, 8 µL of the Matrigel matrix mixture and complete culture medium were used to coat wells of a 384-well plate in advance. A part of the PDO pellets was incubated in 0.25% trypsin-EDTA solution for 10 min in order to dissociate them into single cells. Dissociated cancer cells were then resuspended in complete culture medium containing 2% Matrigel and then seeded onto the precoated 384-well plate (100–200 cells in 22.5 µL per well). The cells were allowed to recover at 37 °C for two days and 2.5 µL of complete culture medium containing 0.1, 1, 10, 100, or 500 µM of gemcitabine, 5-fluorouracil, cisplatin, paclitaxel, infigratinib, or ivosidenib was added into the wells. After incubating for 96 h, cell viability was measured using the CellTiter-Glo 3D Cell-Viability assay (Promega, WI, USA). Wells with complete culture medium containing no drugs served as a negative control. Each drug screening was performed in triplicate.

### Statistical analysis

All statistical analyses were performed using GraphPad Prism software 7.0 (GraphPad Software, CA, USA).

## Results

### Establishment of cancer PDOs

**Fig. 1:**
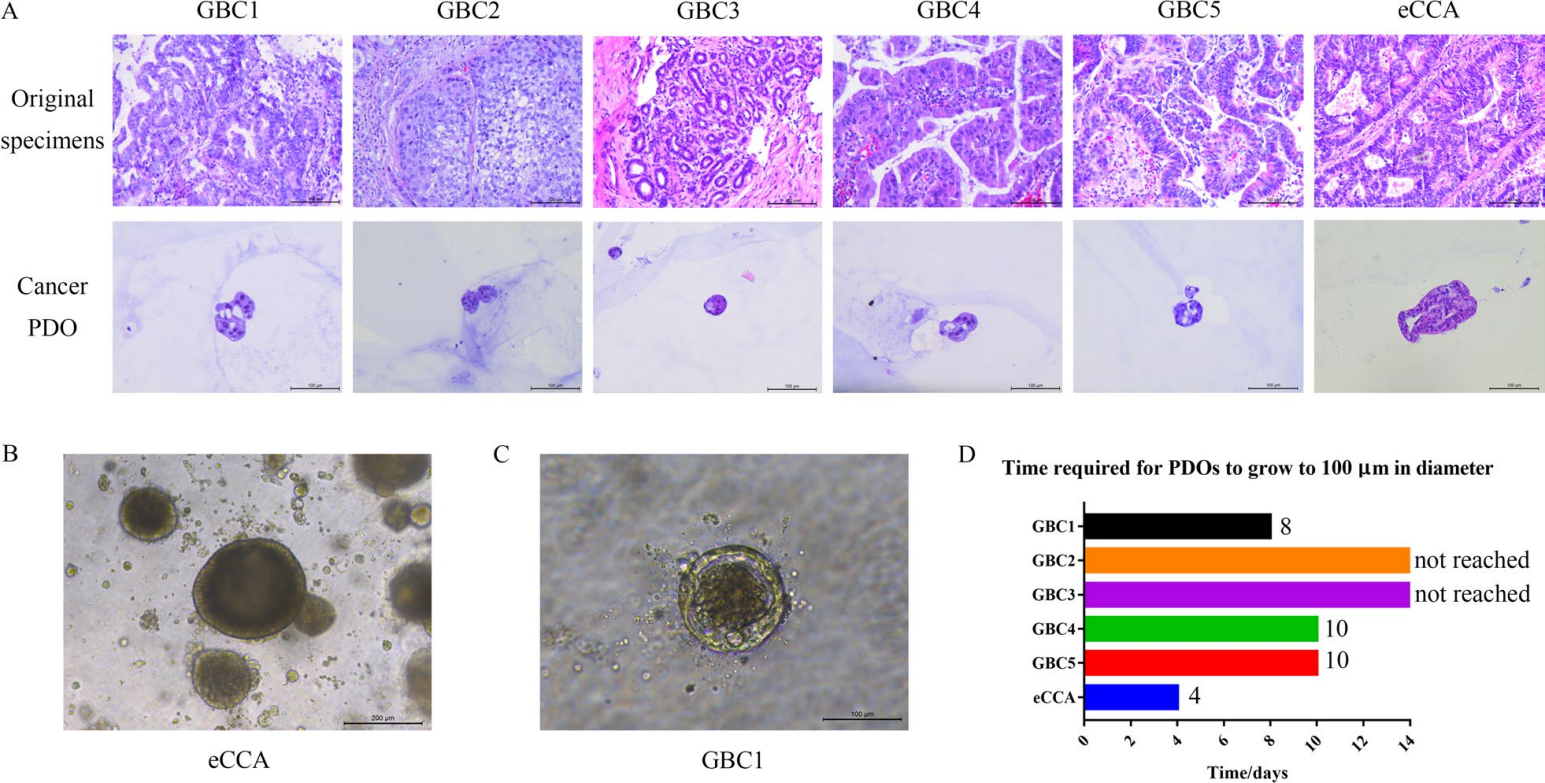
Establishment of eBTC PDO. **A** H&E staining of original specimens and cancer PDOs are presented. Scale bar: 100 μm. **B** The diameter of eCCA PDO reached up to more than 200 μm after the culture of eight days. Scale bar: 200 μm. **C** The diameter of GBC1 PDO reached up to 100 μm after the culture of eight days. Scale bar: 100 μm. **D** The time required by established PDOs to grow to 100 μm is presented

Overall, six GBC specimens were collected, which included five specimens from surgical resection and one specimen from fine needle aspiration. However, the organoid established from the aspiration specimen failed after a two-week culture. In addition, one eCCA specimen was collected from surgical resection. A total of six cancer PDOs were successfully established. According to their H&E staining images, shown in Fig. 1A, most cancer PDOs retained the classic structures of adenocarcinoma. Generally, the larger the cancer PDO, the more similar it was to the original specimen. Indeed, the PDO obtained from the eCCA was the largest and retained most structural features when compared with the original specimen.

Furthermore, the resulting growth rates of cancer PDOs were all different. The mean PDO diameter ranged from 50 to 100 μm. The time required for GBC4 and GBC5 to reach 100 μm was 10 days. The organoid established from the eCCA grew the fastest and its diameter reached over 200 μm after being cultured for eight days, as illustrated in Fig. 1B. Conversely, most PDOs established from GBC specimens grew at a much slower rate than the eCCA PDO. As shown in Fig. 1C, the diameter of the GBC1 PDO reached 100 μm culturing for eight days of culture. Figure 1D summarizes the growth of the six PDOs to 100 μm. We observed that the eCCA PDO rapidly grew up to 100 μm within 4 days, whereas two of the GBC PDOs were not able to reach 100 μm after 2 weeks.

### Histopathological features of cancer PDOs

**Fig. 2:**
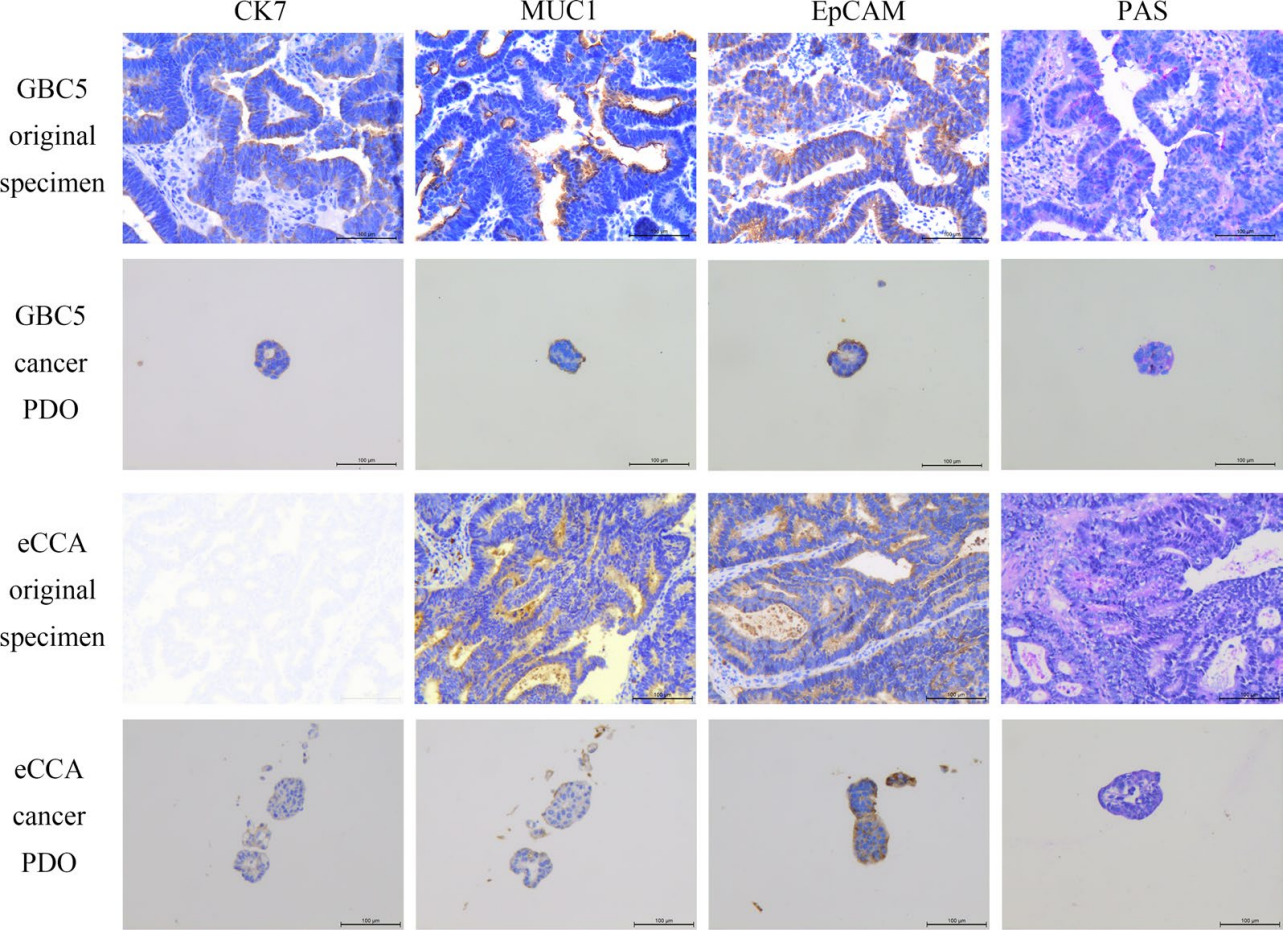
Immunohistological staining and PAS staining of original specimens and PDOs of GBC5 and eCCA. The antigens used in immunohistological staining include CK7, MUC1 and EpCAM. Scale bar: 100 μm

In order to increase the reliability of the subsequent drug screening results, we first needed to prove that cancer PDOs highly retain the structural characteristics of the original specimens. Therefore, immunostaining was carried out to observe histopathological features of cancer PDOs and compare them with the original specimens. CK7, MUC1 and EpCAM were chosen as representative markers because they are frequently expressed in adenocarcinoma. CK7 expression was strong in the original GBC1 and GBC3 specimens but moderate in the original GBC2, GBC4, and GBC5, whereas it was weak in the eCCA (Fig. 2 and Additional file 1: Figure S1). The expression of MUC1 was strong in the original GBC3 specimen, while it was moderate in the original GBC2, GBC4, and GBC5 and weak in the original GBC1 and eCCA specimens (Fig. 2 and Additional file 1: Figure S1). In addition, EpCAM expression was moderate in the original GBC1, GBC5 and eCCA specimens but weak in the original GBC2, GBC3, and GBC4 specimens (Fig. 2 and Additional file 1: Figure S1). Furthermore, we evaluated the distribution of glycogen in the original specimens and cancer PDOs using PAS staining. All specimens except for GBC2 resulted rich in glycogen (Fig. 2 and Additional file 1: Figure S1). When comparing cancer PDOs with their corresponding original specimens, the expression of these selected antigens was consistent and PAS staining also exhibited a similar distribution (Fig. 2 and Additional file 1: Figure S1).

### Genetic profiles of cancer PDOs

**Fig. 3:**
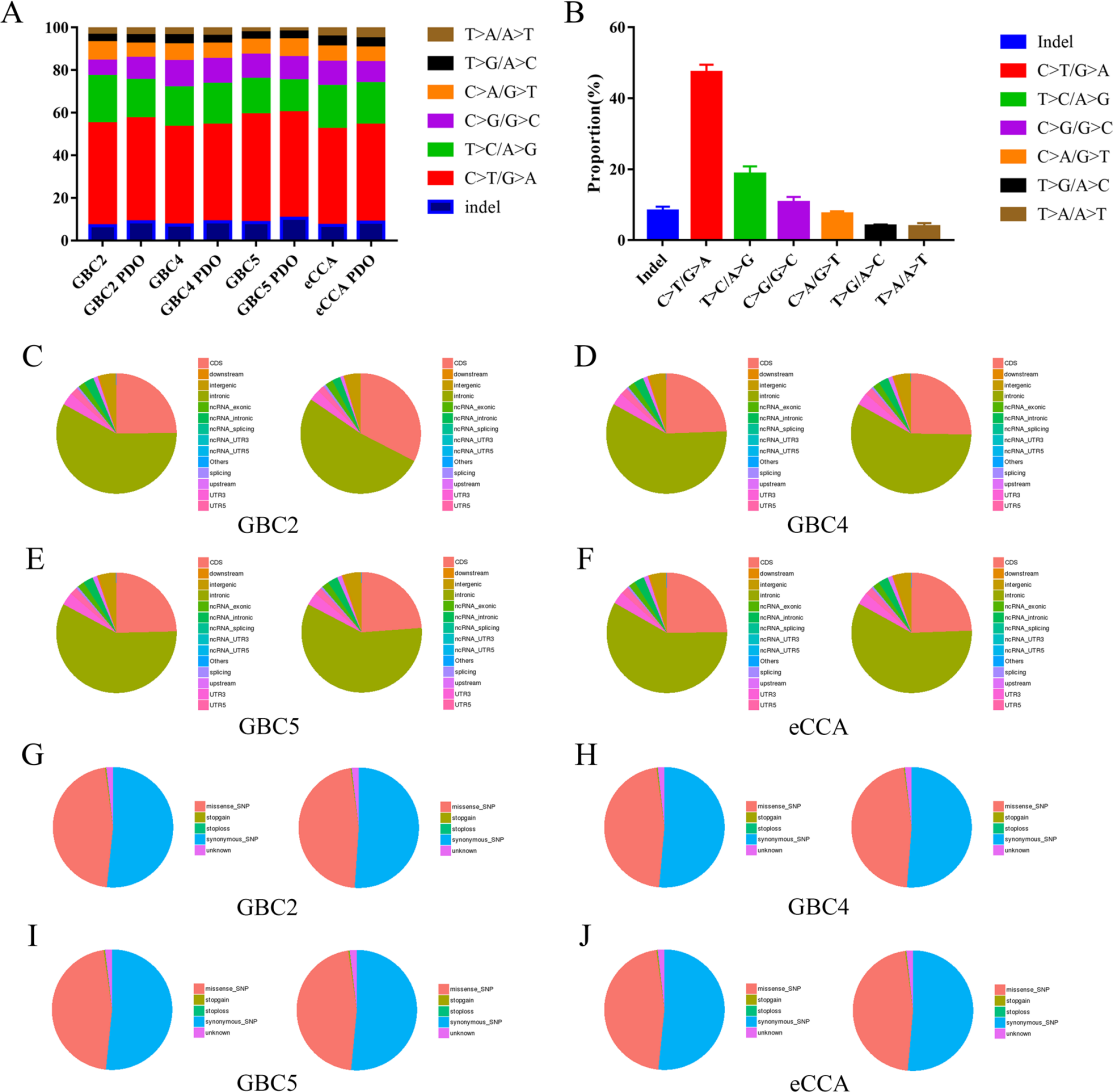
General genetic alterations in four original specimens and cancer PDOs. **A** Proportions of base substitutions and InDels in both specimens and PDOs are represented. **B** Distributions of base substitutions and InDels in all specimens and PDOs are presented. **C**–**F** The numbers of SNPs in different regions of the genome in original specimens (left) and PDOs (right) are presented. The types of regions are shown in the legends. **G**–**J** The distributions of different types of SNPs in coding regions in original specimens (left) and PDOs (right) are presented. The types of SNPs are shown in the legends

To confirm that cancer PDOs retained the genetic profiles of the original specimens, WES was performed using total extracted DNA. We first analyzed the distribution of overall base substitution and the number of SNPs and InDels. We initially compared the distributions of base substitutions, which represent six different types of SNVs, and InDels among PDOs and specimens (Fig. 3A). The results demonstrated that cancer PDOs retained base substitutions and InDels of corresponding specimens in culture. Distributions of the base substitutions and InDels among all PDOs and specimens are summarized in Fig. 3B. Results evidenced an overrepresentation of C>T/G>A transversions, followed by T>C/A>G and C>G/G>C transversions, while the representations of T>A/A>T and T>G/A>C transversions were the least. The number of SNPs in different regions of the genome and the distribution of different types of SNPs in coding regions are shown in Fig. 3C–F, G–J, respectively. We discovered that the proportion of SNPs in the coding sequence was much higher in GBC2 PDO than in the GBC specimen, with a concordance of 74% (Fig. 3C). However, there was a much higher concordance when comparing them across different types of SNPs in the coding region (Fig. 3G). On the contrary, both the number of SNPs in different regions of the genome and distribution of different types of SNPs in the coding region indicated a considerable concordance in the comparisons of the other three cancer organoids and original specimens. Further analysis of InDels across different regions of the genome, as well as different types of InDels in the coding region, indicated that the similarity of InDel was higher than that of SNPs between the original specimens and PDOs (Additional file 2: Figure S2).

**Fig. 4:**
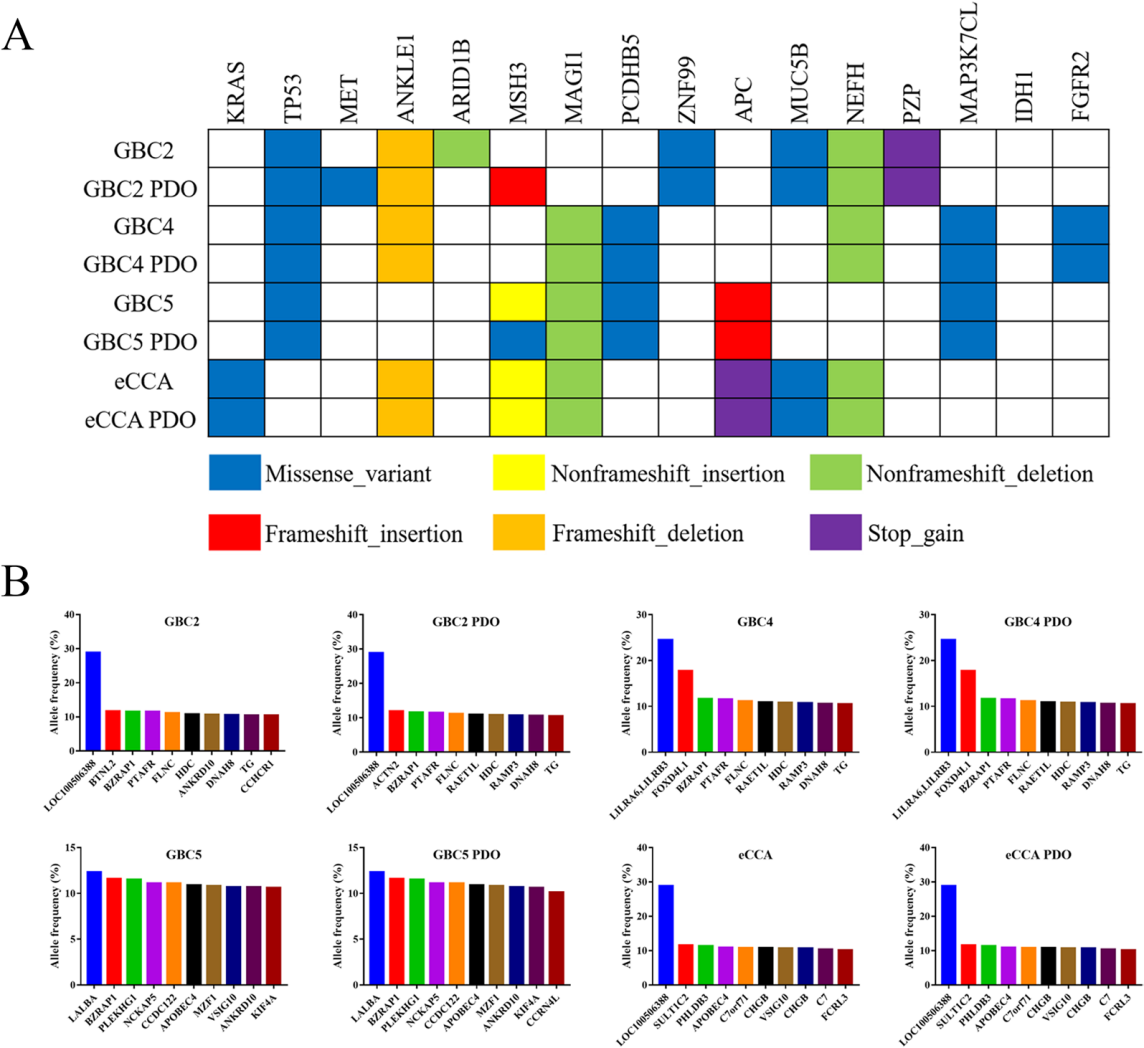
Detailed genetic profiles in four original specimens and cancer PDOs. **A** Some representative genetic variants in specimens and PDOs are presented. The types of variants are shown in the legends. **B** Those variants in specimens and PDOs with an allele frequency of 10–30% in the East Asian population were summarized

Figure 4 A shows a few representative genetic variants to clarify the similarity of specific genes between cancer PDOs and original specimens in detail. Both the eCCA PDO and specimen harbored the *KRAS* missense variant, while other three pairs of PDOs and specimens all harbored the *TP53* missense variant. In general, genetic variants of established PDOs were consistent with those of the original specimens in our study, with the exception of observed differences between the GBC2 PDO and its original specimen in terms of *MET*, *ARID1B*, and *MSH3* variants. Furthermore, we discovered that the GBC5 specimen harbored a *MSH3* non-frameshift insertion, whereas the GBC5 PDO harbored the *MSH3* missense variant. Those variants in PDOs and specimens with an allele frequency of 10–30% in the East Asian population are summarized in Fig. 4B. The GBC2 PDO harbored three different genetic variants in top ten genetic variants, compared with the GBC2 specimen, while the top ten genetic variants are practically the same among other PDOs and specimens.

### Drug screening of cancer PDOs

**Fig. 5:**
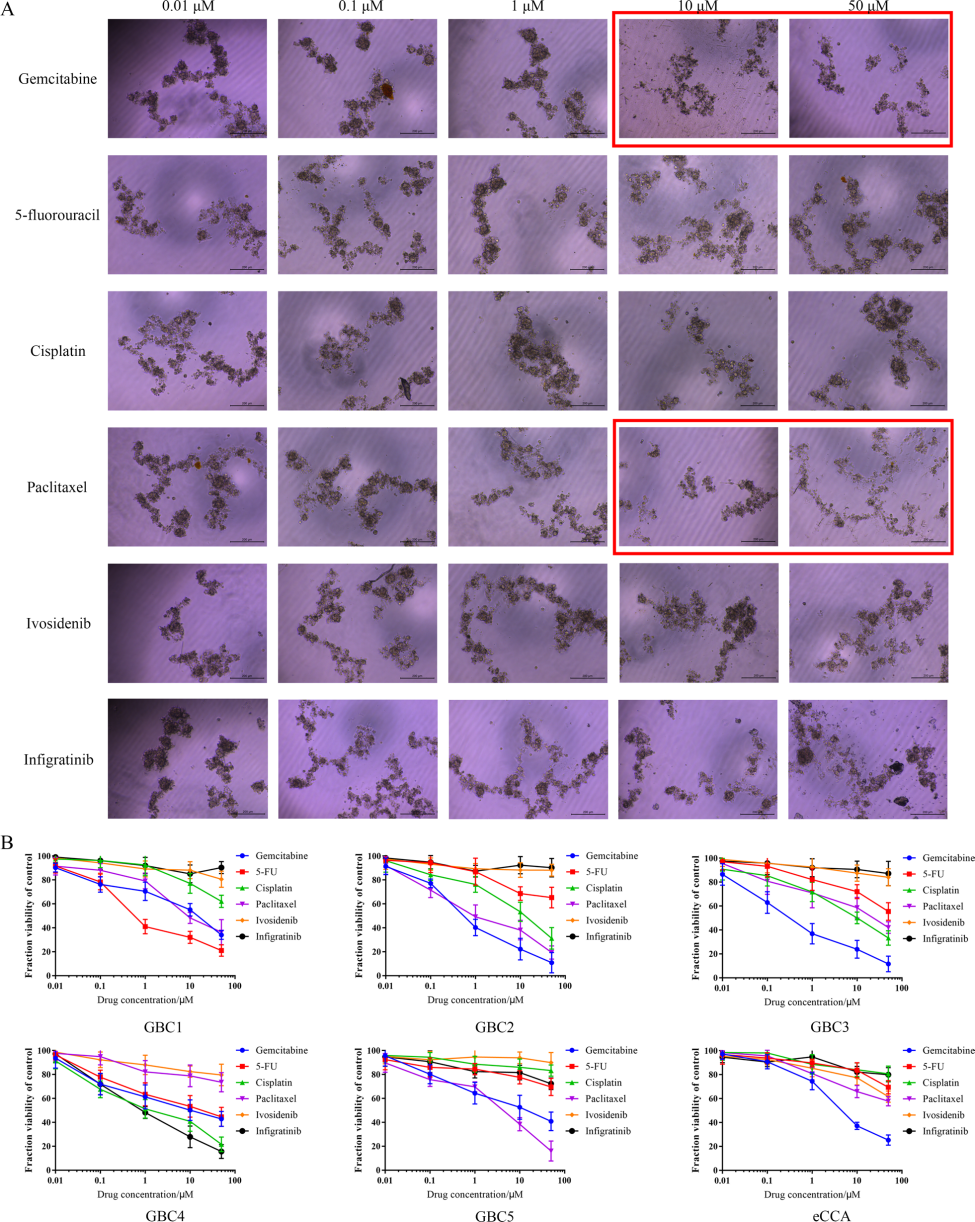
Drug screening in cancer PDOs. **A** Representative images of drug screening in GBC5 PDO are presented. The drugs and concentrations with the strongest anti-tumor effect are marked by the red box. **B** Dose-response curves of all six cancer PDOs are presented. The types of drugs are shown in the legends

After confirming there was a high similarity between PDOs and original specimens, we evaluated drug sensitivity of each PDO by measuring cell viability after drug treatment. The PDOs exhibited differential growth based on the addition of drugs and representative images of drug screening of GBC5 PDO, as shown in Fig. 5A. Our findings revealed that the growth of the GBC5 PDO was significantly inhibited by treatment with 10 or 50 µM paclitaxel. In addition, PDO growth was moderately inhibited by treatment with 10 or 50 µM gemcitabine. Other drugs, including 5-fluorouracil, cisplatin, ivosidenib, and infigratinib, did not significantly affect the growth of the GBC5 PDO. Dose-response curves of GBC5, as well as the other five cancer PDOs, are shown in Fig. 5B. According to our results, gemcitabine was the most efficient drug for eBTCs, as evidenced by its moderate or significant inhibitory impact on cancer growth. Among the three conventional chemotherapy agents, 5-fluorouracil, cisplatin and paclitaxel, can have antitumor effects on some of these cancers. According to WES results, GBC4 harbored the *FGFR2* missense variant, which was accompanied by a significant inhibitory effect of infigratinib on GBC4 PDO during drug screening. The remaining five PDOs did not harbor any *IDH1* or *FGFR2* variants, based on our analysis. Accordingly, ivosidenib and infigratinib did not exhibit noticeable inhibitory effects on these PDOs.

### Comparison with clinical effects

Eventually, the drug sensitivity results for cancer PDOs were compared with actual clinical effects in patients providing original specimens. Therefore, the reliability of drug sensitivity results of cancer PDOs mainly depended on their comparisons with the clinical effects reported for three patients that received adjuvant chemotherapy in this study. Of these patients, the eCCA patient received postoperative adjuvant chemotherapy, while two GBC patients underwent neoadjuvant chemotherapy prior to surgery. Their responses to treatment were recorded in this study.

**Fig. 6:**
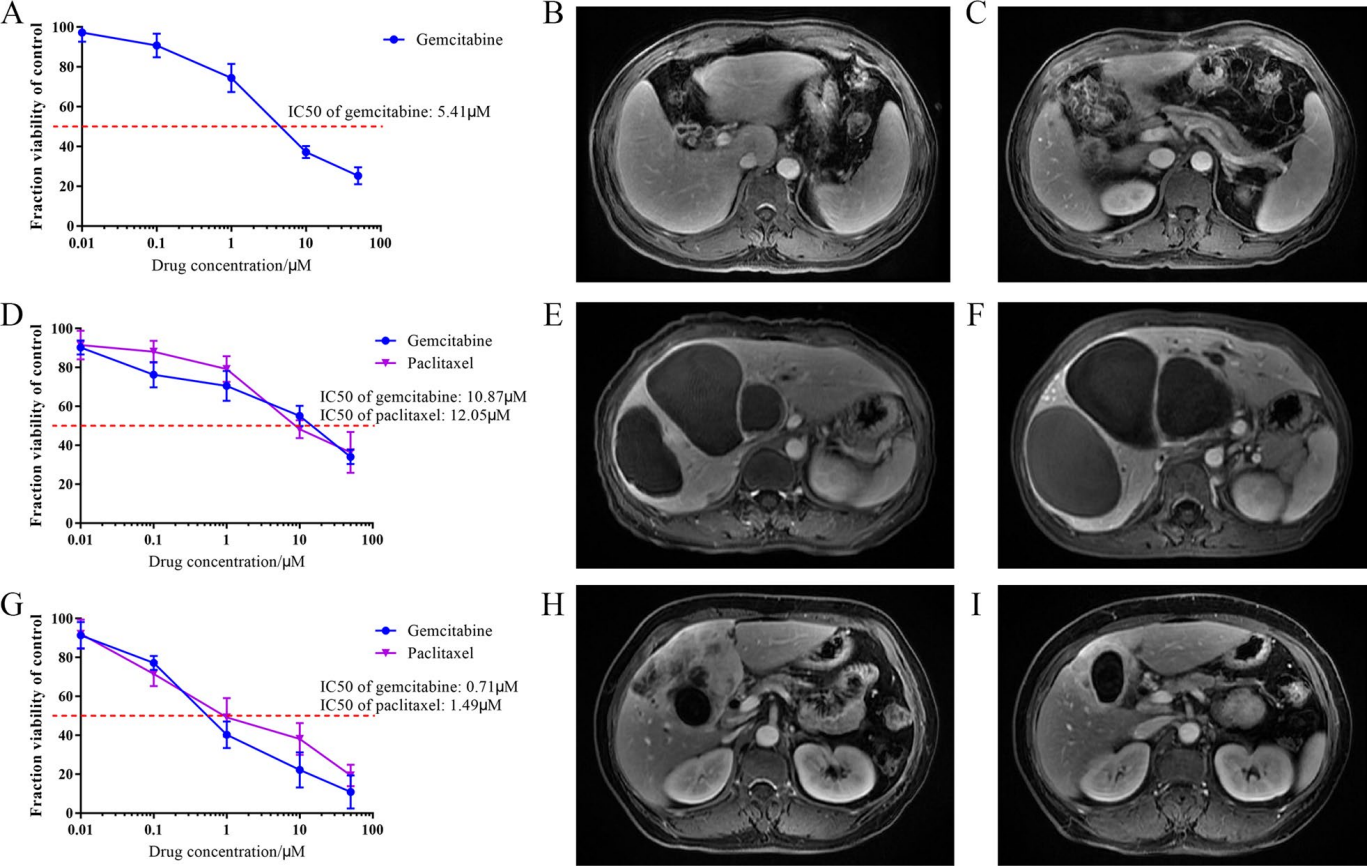
Three patients receiving perioperative adjuvant treatment. **A** Dose-response curve of gemcitabine in eCCA PDO is presented. **B**, **C** The eCCA patient was diagnosed as eCCA on October 8, 2020 according to MR images (**B**) and received surgical resection (**C**). **D** Dose-response curve of gemcitabine and paclitaxel in GBC1 PDO is presented. **E**, **F** The GBC1 patient was diagnosed as GBC on June 11, 2020 according to MR images (**E**). Then gemcitabine and albumin-bound paclitaxel were given for two cycles but MR images indicated the tumor had progressed according to MR images (**F**). **G** Dose-response curve of gemcitabine and paclitaxel in GBC2 PDO is presented. **H**, **I** The GBC2 patient was diagnosed as GBC on August 7, 2020 according to MR images (**H**) and received neoadjuvant chemotherapy of gemcitabine plus albumin-bound paclitaxel plus tislelizumab for 12 cycles, showing partial response (**I**)

Firstly, results from drug screening in eCCA PDO demonstrated that gemcitabine was the most efficient drug for this patient, with a 50% inhibitory concentration (IC50) of 5.41 µM (Fig. 6A). The magnetic resonance (MR) images of the eCCA patient at diagnosis and post-surgery are presented in Fig. 6B, C. The patient was prescribed with post-surgical gemcitabine monotherapy and followed-up for six months, with no recurrence or metastasis observed.

Secondly, 5-FU demonstrated the most significant inhibitory effects on GBC1 PDO (Fig. 5B), while gemcitabine and paclitaxel did not show significant anti-tumor effects in drug screening, as shown in Fig. 6D, with an IC50 concentration of 10.87 and 12.05 µM, respectively. The GBC1 patient underwent neoadjuvant chemotherapy of gemcitabine, plus albumin-bound paclitaxel, for two cycles. However, we observed disease progression, as shown in Fig. 6E, F. Although tislelizumab was then given to the patient for two cycles, it failed to reduce tumor size.

Thirdly, gemcitabine and paclitaxel were the most efficient drugs for GBC2, based on our drug screening (Fig. 6G). Accordingly, after the GBC2 patient received neoadjuvant chemotherapy of gemcitabine plus albumin-bound paclitaxel plus tislelizumab for 12 cycles, the size of tumor decreased by approximately 40% (Fig. 6H, I), indicating a partial response to the treatment.

## Discussion

An increasing number of PDOs derived from different cancers have been established in recent years. However, hardly any studies have been conducted to establish cancer PDOs from eBTC. Our study is the first to collect a series of GBC and eCCA specimens and establish corresponding cancer PDOs. It has been reported that eBTC PDOs are able to retain histopathological features and genetic profiles of the original specimens (i.e., PDOs from other cancers), and predict drug sensitivity of the original specimens to some extent [9–12].

Currently, there are two main methods to obtain clinical specimens. One is through surgical resections, while the other is by obtaining biopsy specimens. Additionally, a few other methods exist, such as obtaining samples from bile and urine [17, 18], although these have not been widely used yet. Herein, we obtained six specimens from surgical resection and one specimen from a fine-needle aspiration biopsy. After culturing them for two weeks, the PDO established from the biopsy specimen failed due to an insufficient amount of cancer cells. Previous studies have established numerous cancer PDOs using biopsy specimens, with a success rate of 63–78% for several years [10, 19]. The protocol of obtaining specimens through a fine-needle aspiration usually requires multiple punctures to obtain enough specimens [20]. However, puncture biopsy, particularly in the liver, is prone to bleeding due to the abundant blood supply. Multiple punctures inevitably lead to a higher risk of bleeding. In our hospital, only one extra specimen can be obtained in addition to routine diagnosis. Hence, the amount of cancer cells is usually insufficient for PDO culture. Nuciforo et al. attempted to established liver cancer PDOs using tumor needle biopsies. Nonetheless, the success rate was found to be solely of 26% [21]. Obtaining sufficient specimens with less trauma remains an urgent problem to be addressed.

Different cancers have their own growth patterns, which is also reflected in the growth of PDOs. Among the PDOs that were established in our study, the eCCA PDO grew at a fastest rate (it was able to reach 100 μm in four days and reached nearly 200 μm within 8 days). The growth of this eCCA PDO halted at day 9, since the growth rate of PDOs significantly slowed down when their diameter reached 150–200 μm. This can be explained by the fact that nutrient requirements increase together with PDO size; hence, the nutrients within the culture medium are no longer sufficient. Additionally, increasing diameter makes it more difficult for cells located in the center of PDO to obtain nutrients, which in turn hampers growth. Thus, it is cost efficient to culture PDOs no larger than 150 μm, which provides enough material to process and further perform histological staining, WES, and drug screening. On the contrary, the PDOs of GBC2 and GBC3 grew at a slower rate and were unable to reach 100 μm within two weeks. Even though prolonging culture time led to an increase in cancer PDO size, a lengthy waiting time is unacceptable for tumor patients undergoing a practical clinical situation. Therefore, our study simulated the actual clinical situation and limited the total culture time to no longer than two weeks.

Different PDOs received the same complete culture medium, as recommended in previous studies [11, 14, 22]. However, different cancers likely harbor different mutations of driver genes, and thus require different essential nutrients. PDOs derived from BTCs with *TP53* and *KRAS* mutations required the addition of nutlin-3a for further culture, while the existence of EGF would decrease the success rate of PDO establishment [23]. The measurement of gene mutations can take a few days to several weeks, and is done using quantitative real-time PCR, DNA sequencing with the Sanger method, or next generation sequencing, which is not acceptable for PDO culture because of its prolonged duration [24–26]. In addition, the relationship between gene mutations and growth factors has not yet been corroborated. Herein, eCCA harbored *KRAS* missense variants, while three GBCs harbored the *TP53* missense variant and ought to grow relatively slower without nutlin-3a. However, they exhibited totally different growth rates when cultured in the same medium without nutlin-3a. From a financial point of view, a set of culture medium formula that is suitable for culturing most BTC PDOs is required. Zhao et al. discovered that lactate promotes the self-renewal of cancer stem cells in colorectal cancer PDOs [27]. Although the underlying mechanisms have not yet been investigated, this provides new insight for potential methods to promote PDO growth rate.

Based on our findings, histological structures of established PDOs maintained the features of adenocarcinoma. However, certain PDOs differ from their corresponding original specimens, as reported in previous studies [15, 28, 29]. A possible explanation is that we were more inclined to digest specimens into a small cluster of cancer cells rather than a single cancer cell. A small cluster of cancer cells allows establishing cancer PDOs more easily and grow faster than single cancer cells [30]. Cancer PDOs from cancer cell clusters are different from the original specimens to some extent, due to compression between adjacent cancer cells, particularly when the size of PDOs is small. Immunohistological staining was then utilized to measure the expression of several biomarkers in PDOs, which included CK7, MUC1, and EpCAM. Our results demonstrated that biomarker expression in established cancer PDOs is similar to that in the original specimens, which indicates that using cell clusters does not affect PDO culture. Pasch et al. identified phenotypic heterogeneity within the same culture of PDOs that were derived from colorectal adenocarcinoma and contained PDOs with lumen and devoid of lumen, due to a mixture of areas with and without glands in specimens [31]. Phenotypic heterogeneity likely exists in PDOs from our study as well and depends on the types of tissues in the specimens.

Cancer PDOs are able to retain genetic profiles of original specimens, even after several passages [10, 32]. The genetic profiles of four eBTC PDOs were measured using WES and compared with those of their corresponding specimens. These results demonstrated that three of the four PDOs retained similar genetic profiles to the original specimens. However, genetic alterations of GBC2 PDOs were diverse compared with the GBC2 specimen, with merely 74% concordance among all SNPs across different regions of the genome. Lee et al. also reported that four of the 15 established bladder cancer PDOs had less than 60% concordance of genetic profiles, when compared with the corresponding specimen [33]. This issue has been raised across multiple studies. Certain cancer PDOs are not able to efficiently recapitulate the structures and genetic profiles of original specimens due to a lack of the necessary microenvironment [34, 35]. Interestingly, there is also significant heterogeneity between the same types of cancer. Poorly differentiated and highly invasive cancer cells are able to grow into organoids, even without the assistance of stromal cells, while other well differentiated cells have genetic variants due to a lack of certain cytokines or cellular interaction. A study recently reported that co-culture of cancer-associated fibroblasts and liver cancer cells promoted growth of cancer PDOs [36]. In practice, controlling the number of stromal cells in the co-culture system is essential, since stromal cells grow faster than tumor cells, and an excessive number of stromal cells would occupy the living space of tumor cells.

Four chemotherapeutic drugs, as well as two targeted drugs, were utilized for drug screening in our study. The dose-response curves, acquired from all six cancer PDOs, indicated that gemcitabine was the most efficient drug for eBTC treatment, showing moderate or significant inhibitory impact on the growth of PDOs. It can be inferred that abnormal expression of proteins related to gemcitabine resistance in established PDOs, such as equilibrative nucleoside transporter, deoxycytidine kinase, ribonucleotide reductase M1 or M2, and ATP-binding cassette transporter family, was lower than that of other proteins related to the resistance of 5-fluorouracil, cisplatin, and paclitaxel according to WES results. Results from drug screening indicated that the most effective drug for the enrolled eCCA patients was gemcitabine, with a 50% inhibiting concentration of 5.41 µM. The eCCA patient was treated with gemcitabine monotherapy post-surgery and had no recurrence or metastasis for six months. However, it was difficult to validate the predictive role of cancer PDOs in drug susceptibility among the surgically resected patients, due to the many factors that affect recurrence and metastasis after surgery, including tumor stage, metastasis, margin status, and vascular invasion [37]. Therefore, eCCA patients need to be followed up for longer periods of time. Conversely, the GBC1 patient initially received neoadjuvant chemotherapy with gemcitabine, plus albumin-bound paclitaxel, but disease progression was observed after two cycles. The patient then received extra tislelizumab for two cycles but failed to control the progression of cancer again. According to the dose-response curves, 5-FU was the most effective drug for GBC1, followed by gemcitabine and paclitaxel, with a high concentration of IC50, which was consistent with the observed clinical effects. Furthermore, the GBC2 patient received neoadjuvant chemotherapy with gemcitabine plus albumin-bound paclitaxel plus tislelizumab for 12 cycles and experienced a partial response after treatment. This is consistent with the results obtained from the dose-response curves, in which gemcitabine and paclitaxel acted as the most two effective drugs for GBC2. However, this does not fully account for the predictive role of PDOs in drug susceptibility, as these patients were also treated with tislelizumab. Tislelizumab is an immune checkpoint inhibitor that targets programmed cell death protein 1 and is able to activate lymphocytes to kill cancer cells. In order to screen the drug susceptibility of tislelizumab in PDOs, the tumor immune microenvironment needs to be constructed. Nevertheless, only a few studies have successfully constructed the tumor immune microenvironment in cancer PDOs. Neal et al. reported a novel air-liquid interface method for co-culturing cancer PDOs and native tumor-infiltrating lymphocytes [38]. This method allows to determine the *in vitro* effect of immune checkpoint inhibitors on cancer [38]. We also attempted to replicate this method and discovered that stromal cells can easily occupy the living space of cancer cells, as the specimens had difficulty being digested and a large number of stromal cells were retained. Another method proposed by Dijkstra et al. was co-culture of the peripheral blood lymphocytes and cancer PDOs to construct the immune microenvironment [39]. However, this method can inhibit the growth of PDOs, as the lymphocytes will kill the cancer cells. Therefore, more attempts are needed to improve the method of preserving the immune microenvironment.

Our study has certain limitations. First, the aim of our study was to establish cancer PDOs of eBTC. However, only one specimen of eCCA was collected for PDO establishment. Considering that eBTC includes both GBC and eCCA, more eCCA specimens are needed to further confirm the efficacy of eBTC PDO establishment. Since specimens were collected from a single center in this study, collecting more eCCA specimens proved difficult due to its low incidence. A multicenter study may be a better option to solve this problem. Second, two DNA samples of GBC PDOs were degraded due to poor preservation, leading to incomplete WES results, as well as insufficient analysis of concordance between cancer PDOs and original specimens. Third, only six drugs were included in the drug screening of PDOs due to a relatively small quantity of PDOs after culturing for two weeks. Fourth, established PDOs in this study lacked the immune microenvironment, which plays a crucial role in tumor progression and drug sensitivity.

Moreover, the significance of this study was to demonstrate the feasibility of PDO establishment from eBTC and the predictive value of PDO in cancer treatment. This study provided important evidence for clinical applications of PDOs, as well as pinpointed relevant issues that need to be addressed in future studies.

## Conclusions

In summary, eBTC PDOs were successfully established from five GBC specimens and one eCCA specimen in this study, which enabled us to fill the existing gap in the field of eBTC PDOs. Results from drug screening in PDOs may provide helpful assistance in selecting chemotherapy drugs or targeted drugs for cancer patients. Further improvements are required in order to improve the growth rate of PDOs and to preserve their immune microenvironments.

## Supplementary Information

**Additional file 1: Figure S1.** Immunohistological staining and PAS staining oforiginal specimens and PDOs of GBC 1-4. The antigens used in immunohistologicalstaining included CK7, MUC1 and EpCAM. Scale bar: 100μm.

**Additional file 2: Figure S1.** The numbers of InDels in different regions of thegenome (A-D) and the distributions of different types of InDels in codingregions (E-H) in original specimens (left) and PDOs (right) are presented. Thetypes of regions and InDels are shown in the legends, respectively.

## Data Availability

All data generated or analyzed during this study are included in this published article and its a information files.

## Abbreviations

CNV: Copy number variation
eBTC: Extrahepatic biliary tract carcinoma
eCCA: Extrahepatic cholangiocarcinoma
GBC: Gallbladder carcinoma
H&E: Hematoxylin and eosin
IC50: 50% inhibitory concentration
iCCA: Intrahepatic cholangiocarcinoma
InDel: Insertions and deletions
PAS: Periodic acid-schiff
PDO: Patient-derived organoid
SNP: Single nucleotide polymorphism
SNV: Single nucleotide variation
WES: Whole exome sequencing
