# Supplementary figures and images for: Establishment and drug screening of patient-derived extrahepatic biliary tract carcinoma organoids
Source: Cancer Cell Int. 2021 Oct 2;21:519. doi: 10.1186/s12935-021-02219-w (PMC8487492; doi:10.1186/s12935-021-02219-w)

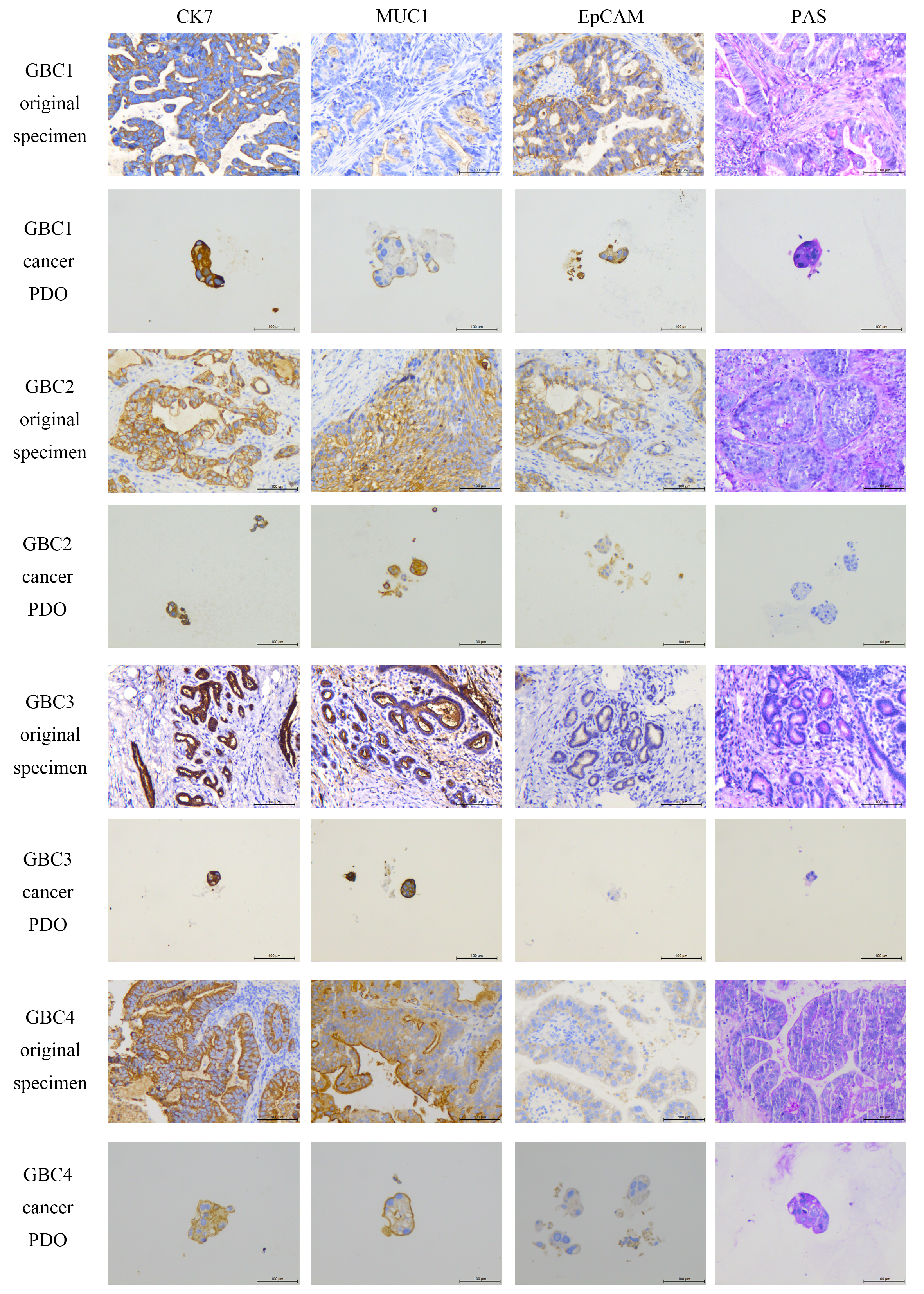

Supplement: Supplementary file 1 — Additional file 1: Figure S1. Immunohistological staining and PAS staining oforiginal specimens and PDOs of GBC 1-4. The antigens used in immunohistologicalstaining included CK7, MUC1 and EpCAM. Scale bar: 100μm. [file 12935_2021_2219_MOESM1_ESM.tif]

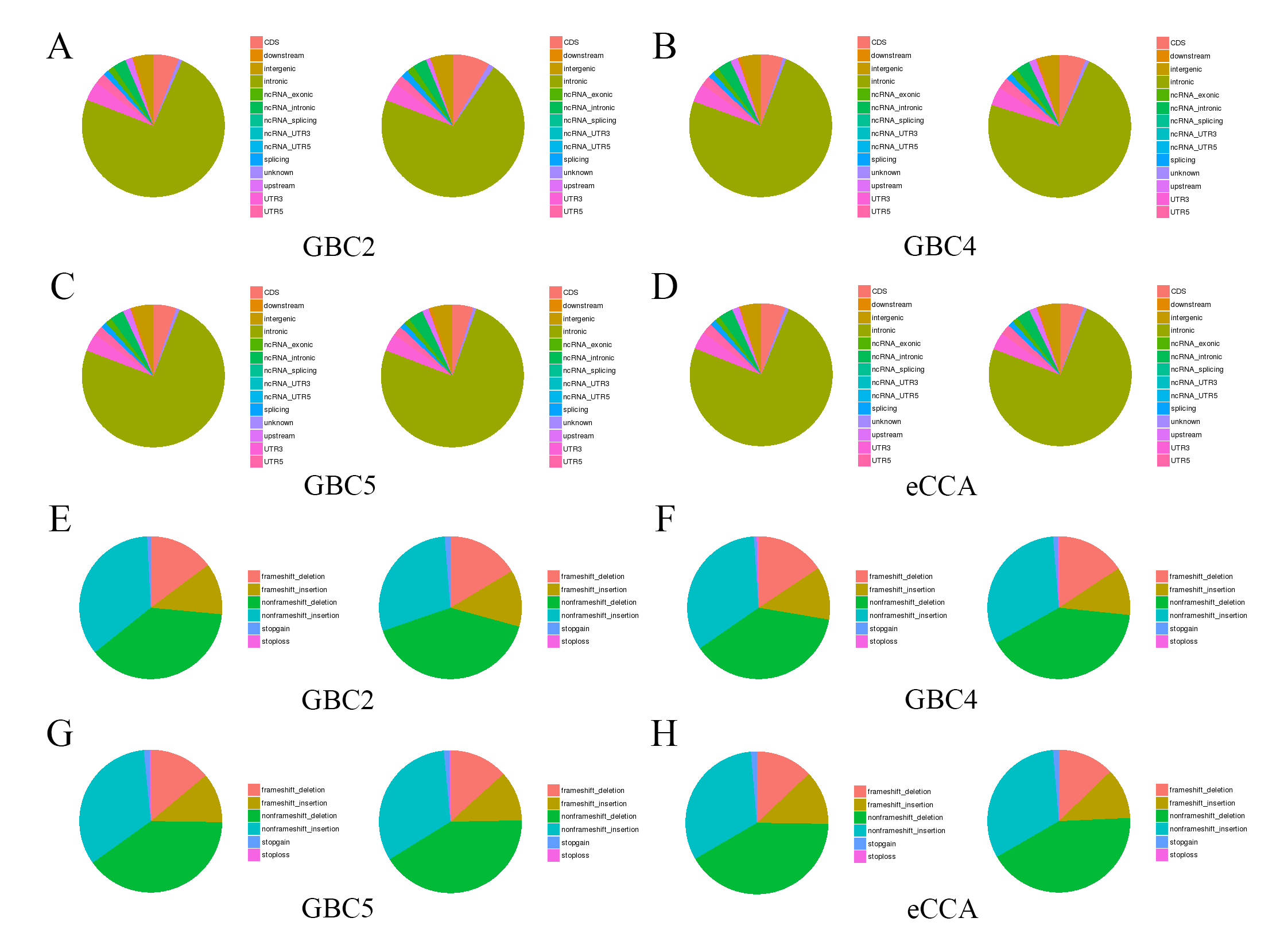

Supplement: Supplementary file 2 — Additional file 2: Figure S1. The numbers of InDels in different regions of thegenome (A-D) and the distributions of different types of InDels in codingregions (E-H) in original specimens (left) and PDOs (right) are presented. Thetypes of regions and InDels are shown in the legends, respectively. [file 12935_2021_2219_MOESM2_ESM.tif]
